# Climate warming and Bergmann's rule through time: is there any evidence?

**DOI:** 10.1111/eva.12129

**Published:** 2013-11-25

**Authors:** Celine Teplitsky, Virginie Millien

**Affiliations:** 1 Département Ecologie et Gestion de la Biodiversité UMR 7204 CNRS/MNHN/UPMC, Muséum National d'Histoire NaturelleParis, France; 2 Redpath Museum, McGill UniversityMontreal, QC, Canada

**Keywords:** adaptation, animal model, Bergmann's rule, birds, body size, climate change, mammals, microevolution, natural selection, phenotypic plasticity

## Abstract

Climate change is expected to induce many ecological and evolutionary changes. Among these is the hypothesis that climate warming will cause a reduction in body size. This hypothesis stems from Bergmann's rule, a trend whereby species exhibit a smaller body size in warmer climates, and larger body size under colder conditions in endotherms. The mechanisms behind this rule are still debated, and it is not clear whether Bergmann's rule can be extended to predict the effects of climate change through time. We reviewed the primary literature for evidence (i) of a decrease in body size in response to climate warming, (ii) that changing body size is an adaptive response and (iii) that these responses are evolutionary or plastic. We found weak evidence for changes in body size through time as predicted by Bergmann's rule. Only three studies investigated the adaptive nature of these size decreases. Of these, none reported evidence of selection for smaller size or of a genetic basis for the size change, suggesting that size decreases could be due to nonadaptive plasticity in response to changing environmental conditions. More studies are needed before firm conclusions can be drawn about the underlying causes of these changes in body size in response to a warming climate.

## Introduction

Biological responses to global change fall into three categories: extinction, change in distribution and adaptation (genetic or plastic) to the new environmental conditions. Alterations in species distribution and abundance that track the shifting climate have been widely documented in a number of plant and animal species (Parmesan and Yohe 2003; Root et al. 2003). Similarly, there is a reasonable amount of evidence for the relation between climate warming and changes in the phenology of species (Stenseth et al. 2002; Walther et al. 2002; Bradshaw and Holzapfel 2006; Parmesan 2006; Boutin and Lane 2014; Charmantier and Gienapp 2014). Recently, changes in body size associated with climate change have also received growing interest in the context of current climate warming (Gardner et al. 2011; Sheridan and Bickford 2011).

Body size is known to vary with latitude in a number of taxa, from invertebrates to endotherm species. The tendency of individuals within the geographical range of a species to be larger in body size under colder climatic conditions is known as Bergmann's rule (Bergmann 1847; Mayr 1956), and is best supported in endotherms, namely birds and mammals (Ashton 2002; Meiri and Dayan 2003; Millien et al. 2006). For example, a trend of increasing body size with latitude has been found in 65% to 71% of mammals and 72% to 76% of birds (Meiri and Dayan 2003; Millien et al. 2006). A corollary to Bergmann's rule is the trend of increasing body size with altitude. Bergmann's and the altitude rules are often associated, based on the assumption that the underlying mechanism of both these geographical trends has a thermoregulatory basis. The increase in body size would be a response to a decrease in temperature with latitude or altitude, respectively.

A decrease in size with climate warming has been observed in a number of species, over large time scales (reviews in Millien et al. 2006; Blois et al. 2008). In these studies, the change in size was correlated with a change in the environment, a temperature increase in most cases. There is thus a decent amount of data providing support for the relation between climate warming and decrease in size over large time scales (e.g. since the Last Glacial Maximum 20,000 years ago). Such a temporal trend is expected in analogy with its geographical equivalent, Bergmann's rule. In theory, a comparison across different geographical and temporal scales can be achieved by studying the relations between a trait such as body size and an environmental factor such as temperature (Polly et al. 2011). It is assumed that the functional relation between this trait and the environmental factor does not vary across scales. In other words, the direction and extent of trait variation observed across the geographical range of a species are expected to be similar through time, provided the driving environmental factor varies to a similar extent in both space and time. Given the general nature of Bergmann's rule in endotherms, a decrease in size in these organisms is expected with ongoing climate warming. Accordingly, it has been suggested by some that data from the past could help at better predicting the future response of species to climate warming (Berteaux et al. 2006; Millien et al. 2006; Chown et al. 2010; Kerr and Dobrowski 2013).

A decrease in body size in response to climate warming has recently been deemed as the third response (along with changes in distribution and phenology) to global change (Gardner et al. 2011). A decrease in size over the last decades has been reported in a number of organisms (Gardner et al. 2011; Sheridan and Bickford 2011). It is often assumed that morphological changes occurring in the context of climate change are adaptive, microevolutionary responses, that allow species to better cope with the ongoing climate warming. However, such an interpretation of phenotypic trends relies on two currently unaddressed questions: (i) are these phenotypic changes adaptive? and (ii) if so, are they due to evolution or plasticity?

To address the first question, we need to establish if a decrease in size under a warmer climate improves fitness. The main adaptive mechanism for Bergmann's rule is a decrease in the surface area to volume ratio, reducing heat loss in colder conditions. However, the selection pressure and underlying mechanisms driving Bergmann's rule vary across different groups of animals (Blackburn et al. 1999; Meiri 2011; Olalla-Tarraga 2011; Watt and Salewski 2011). Bergmann's rule can thus be considered both *sensu stricto* and *sensu lato*. In both, a decrease in size is related to an increase in temperature. With a narrow definition of Bergmann's rule, body size decrease is mechanistically linked to energy and heat dissipation, and is adaptive. In a broader sense, the decrease in size may be driven by other factors (e.g. food availability, length of the growth season) than temperature. With that latter definition, size declines may still be adaptive, but not necessarily. Assessing the adaptive nature of size changes thus does not validate Bergmann's rule either way, but is important for assessing the mechanisms that can enhance population persistence in the context of global change. Inferring the direction of selection is also necessary to evaluate the adaptive nature of size changes, whether selection pressures are due to climatic or other environmental variables.

The second question relates to the nature of the size changes, namely whether they are plastic or evolutionary. Adaptive plasticity is a short-term response to environmental changes: organisms express different phenotypes depending on environmental conditions, but it does not involve genetic changes. These different phenotypes are expected to confer higher fitness of individuals in the environment they are expressed in. In turn, microevolution is a longer term response to environmental change. It involves a change in the genetic composition of the population, resulting from directional selection on phenotypes (and thus on genetic variation). There is increasing awareness, however, that microevolution can be fast (Hendry and Kinnison 2001; Hairston et al. 2005), and rapid evolution in response to global change could occur (Berteaux et al. 2004; Bradshaw and Holzapfel 2006). As both microevolutionary and plastic responses can occur rapidly, there is a risk of confounding the two when studying phenotypic changes through time (Gienapp et al. 2008; Hendry et al. 2008). Hence, caution must be used when interpretating temporal phenotypic trends to avoid adaptive storytelling (Gould and Lewontin 1979). Therefore, we need to establish the adaptive nature of size changes and their plastic or genetic basis (Gienapp et al. 2008; Hansen et al. 2012; Merilä and Hendry 2014).

Here, we review the evidence that (i) decreases in body size through time have occurred with recent climate warming in accordance with Bergmann's rule, (ii) these size trends are adaptive in nature, with smaller individuals being selected and (iii) these trends have either a plastic or a genetic basis.

## Evidence for Bergmann's clines in response to climate change

### Reviewing the trends in natural populations

We first assessed the prevalence of patterns that fit Bergmann's rule in time, that is, a direct correlation between body size and temperature, or more broadly, a temporal trend of decrease in body size in a locality where temperature had been increasing. We performed a search in ISI Web of Science. As this search was aimed at evaluating the conclusions of studies investigating Bergmann's clines and not size changes in general, the search was limited to the keywords ‘Bergmann + Climate Change’ OR ‘Bergmann + Global warming’. This search brought 105 references. We retrieved two more references with an additional search performed in Google Scholar using the keywords ‘body size + climate warming’, limited to the last 5 years. Of the 107 studies we found, we excluded papers focusing on past climate changes at a paleontological scale (i.e. predating recent climate warming) as well as studies focusing on geographical clines, thus focusing solely on studies reporting temporal size trends within a population over the last century. This resulted in 22 references (Table S1). For each of these papers, we then assessed whether the detected trends were in agreement with Bergmann's rule, and if these size trends were correlated with temperature.

Overall, there was mixed evidence for Bergmann's rule in response to recent climate warming (Tables 1 and S1). When considering all trait and species combinations, patterns differed between birds and mammals (Fisher's exact test: *P* < 0.01). Patterns in support of Bergmann's rule were found in 60% of the 566 cases in birds, but only in 7% of the 79 cases in mammals. This is in line with Gardner et al. (2011) who emphasized the heterogeneity of responses to climate warming among species. Mammals and birds may respond differently to environmental changes. In fact, Yom-Tov and Geffen (2011) already noted such a discrepancy between birds and mammals. Negative trends in birds were often interpreted as adaptation to climate change following Bergmann's rule's original mechanism (Yom-Tov 2001), whereas positive trends in mammals were thought to reflect an increase in food abundance due to human activities (e.g. Yom-Tov et al. 2006a, 2010b). However, the actual mechanisms and selection pressures underlying the shifts in size were not investigated in these studies.

**Table 1:** Summary of reported temporal trends over 22 studies. ‘Count’ refers to the overall number of trends reported over all traits and species in the considered study, the number in parentheses are for cases focusing on body mass. Studies with an asterisk (*) investigated selection and/or genetic trends. Further details are available in Table S1

			Temporal size trend		
			
	Reference	Count	Decrease	Increase	Not significant
Mammals	Eastman et al. (2012)	6	0	2	4
	Koontz et al. (2001)	1 (1)	0	0	1 (1)
	Meiri et al. (2009)	52	3	3	46
	Ozgul et al. (2009)*	1 (1)	1 (1)	0	0
	Ozgul et al. (2010)*	1 (1)	0	1 (1)	0
	Yom-Tov et al. (2003)	8	0	2	6
	Yom-Tov and Yom-Tov (2005)	4 (1)	0	2 (0)	2 (1)
	Yom-Tov et al. (2006a)	1	0	1	0
	Yom-Tov et al. (2008)	1	0	1	0
	Yom-Tov et al. (2010a)	2	2	0	0
	Yom-Tov et al. (2010b)	2 (1)	0	2 (1)	0
Birds	Gardner et al. (2009)	8	6	0	2
	Goodman et al. (2012)	13 (6)	0	10 (3)	3 (3)
	Husby et al. (2011)*	6 (3)	3 (3)	2	1
	McCoy (2012)	6	3	0	3
	Moreno-Rueda and Rivas (2007)	10 (2)	1 (0)	2 (0)	7 (2)
	Teplitsky et al. (2008)*	1 (1)	1 (1)	0	0
	Van Buskirk et al. (2010)*	490 (245)	309 (197)	26 (0)	155 (48)
	Yom-Tov (2001)	9 (5)	6 (4)	0 (1)	3 (0)
	Yom-Tov et al. (2002)	2 (1)	1 (1)	0 (0)	1 (0)
	Yom-Tov et al. (2006b)	21 (7)	10 (6)	11 (1)	0 (0)
Total	Mammals	79 (5)	6 (1)	14 (2)	59 (2)
	Birds	566 (270)	340 (212)	51 (5)	175 (53)

Body size can be estimated from several metrics, such as wing length, skull dimensions or body mass. (e.g. Damuth and McFadden 1990; Yom-Tov and Geffen 2011). In many of the studies reviewed here, multiple traits were investigated within the same population, and the majority of these studies revealed different trends depending on the trait investigated. This suggests that the choice of metrics has a strong impact on the type of response observed. For example, some linear measurements such as tail, foot or tarsus length are expected to decrease under colder climate to limit heat loss in body extremities (Allen's rule, Allen 1877), opposing Bergmann's rule. Sexual selection is also known to influence body size, or the size of some specific morphological traits in many species (Andersson 1994). Conflicting selection pressures may thus act on different sets of morphological traits. Blackburn et al. (1999) advocated using mass preferentially, as it is a better reflection of the overall body size. When we restricted our study to those investigating body mass, we still found differences between birds and mammals (Fisher's exact test *P* < 0.01). Evidence for size declines was stronger in birds (80% of cases) but still weak in mammals (1 / 5, 20% of cases). Yet, most of these studies did not report the actual slope estimates for the relations between size and time or temperature. Thus, we could not formally test for an effect of the trait investigated on the likelihood of detecting a temporal trend in that trait.

With these data, it remains difficult to evaluate the generality of temporal size changes. Meiri et al. (2009) found little evidence of any change in size in 22 species of carnivorous mammals. Building on the lack of pattern in their study, the authors suggested that there was in fact a potential publication bias towards studies reporting only significant trends. In their study, however, sexual dimorphism was present in a majority of the species considered (Appendix S3 in Meiri et al. 2009) and could have masked a temporal trend. For instance, Post et al. (1999) found that sexual dimorphism resulted in divergent temporal size trends in the red deer in Norway. Males increased while female decreased in size in response to direct and indirect effects of climate warming over the last 30 years. Altogether, data are still lacking to assess the overall prevalence and direction of temporal size trends in the context of recent global change (Meiri et al. 2009; Gardner et al. 2011).

### Mechanisms and predictions in the context of climate change

Bergmann's rule was coined over 150 years ago and despite an extensive body of research accumulated on the topic, the mechanisms behind Bergmann's rule are still controversial (Blackburn et al. 1999; Olson et al. 2009; Meiri 2011; Olalla-Tarraga 2011; Watt and Salewski 2011). The original definition by Bergmann involved a heat preservation mechanism through the reduction of the surface area to volume ratio.

The experiment by Barnett and Dickson (1984) is in favour of a direct effect of temperature on size. Two lines of wild house mice (*Mus musculus*) were reared under two ambient temperature conditions. After 11 generations, adult males of ‘Eskimo mice’ kept at −3°C were nearly 30% heavier than control mice kept at 21°C. There was no replicate in this experiment, yet the work of Barnett and Dickson (1984) constitutes one of the rare direct tests of Bergmann's rule in a laboratory setting for a mammal.

The hypothesis of a heat preservation mechanism has received mixed support (Blackburn et al. 1999; Stillwell 2010), and alternative mechanisms have been proposed to explain the observed latitudinal size patterns. First, in addition to a decrease in the surface area to volume ratio, an increase in body mass also leads to a decrease in metabolic rate and associated energy loss. Second, body size could be affected by multiple climatic factors, including temperature and humidity. Finally, it has been proposed that primary plant productivity (Ho et al. 2010) or food availability could be the main drivers of body size variation in some species. Indeed, there is some evidence that prey size availability can constrain body size in predatory mammals. Larger bodied individuals may also have an advantage at higher latitudes because they can better withstand starvation (Cushman et al. 1993). Recently, McNab (2010) further emphasized the role of food availability by combining different geographical clines in body size under a common ‘resource rule’. Clearly, the debate on the mechanisms underlying Bergmann's rule is not settled (e.g. Blackburn et al. 1999; Yom-Tov and Geffen 2011), and body size is most likely affected by a number of correlated environmental factors.

In the context of climate change, each of these different mechanisms could lead to contrasting responses in body size. Under the heat preservation hypothesis where a colder climate leads to an increase in body size, we would predict individuals to decrease in size with increasing temperature. In contrast, under the starvation resistance hypothesis where a larger body size increases resistance to starvation, we would expect an increase in size because temperature and rainfall are expected to become more unpredictable in the future (Goodman et al. 2012). If Bergmann's rule was in part linked with gradients in food abundance or quality, then predicting the impact of climate change on body size becomes challenging, as this impact will depend on the responses of each individual compartments of the ecosystem (e.g. resources, consumers and predators, Sheridan and Bickford 2011). The inability to make simple, clear predictions about body size changes in response to climate change further emphasizes that the adaptive nature of a size change cannot be inferred from patterns only (conformity or not to Bergmann's rule), and that estimating selection pressures acting on size is key to understand these trends.

## The nature of temporal size trends

### Evidence for adaptive size changes

Given the complexity of mechanisms driving body size variation, the adaptive nature of size changes needs to be evaluated for each specific case, if we are to understand their evolutionary consequences on population dynamics and persistence. Demonstrating the adaptive nature of climate-induced size changes requires an assessment of how much fitness increases with this phenotypic change under new environmental conditions, that is, an assessment of how climate influences patterns of natural selection. As emphasized by Merilä and Hendry (2014), many methods to quantify selection, such as resurrection and reciprocal transplants, cannot easily be implemented with birds and mammals. To date, one of the most powerful tools to investigate the adaptive nature of responses in wild bird and mammal populations is the analysis of natural selection in long-term individual-based longitudinal data sets (Clutton-Brock and Sheldon 2010). Such data allow the assessment of phenotypic selection on multiple traits, by regressing fitness (e.g. survival and / or fecundity) against phenotypic traits (Lande and Arnold 1983). More recently, Coulson and Tuljapurkar (2008) showed that a derivation of the Price equation (Price 1970) in age structured populations can be used in a longitudinal data set to assess not only the part of the trait change due to selection through change in survival and fecundity, but also the contribution of other processes in a variable environment (e.g. change in growth rate, plasticity).

So far, the evidence for the adaptive nature of size changes is weak and selection is rarely estimated in studies investigating climate-related size declines (Table 2). In our review, three studies showed a size decline and investigated selection on size using long-term data with individually marked individuals. None of these studies found evidence of selection for smaller size. In red billed gulls (*Chroicocephalus scopulinus*), no selection on body mass was detected over 40 years (Teplitsky et al. 2008). In contrast, selection on size (tarsus length or body mass) in great tits (*Parus major*) over 29 years was mostly positive (or non significant, depending on the population), and there was no evidence for a temporal change in selection during the study period (Husby et al. 2011). Similarly, in Soay sheep (*Ovis aries*), selection for body mass was positive (Wilson et al. 2007; Gratten et al. 2010) but selection explained only a small fraction of body mass variation over the last 20 years (Ozgul et al. 2009). The lack of significant selection for smaller size in these systems is sufficient to exclude the possibility that the size decrease is adaptive. This is not to say that shifts in body size are always nonadaptive. For instance, in the yellow-bellied marmot (*Marmota flaviventris*), an increase in size observed since 2000 was associated with increased survival and breeding success in the later years of the study, resulting in an abrupt increase in population size (Ozgul et al. 2010). Van Buskirk et al. (2010) suggested the possibility of selection for smaller size in bird species showing a size decrease. In this case, selection on size was estimated by comparing measurements in autumn and the following spring for spring migrants, and averaged across all years. They found some evidence for negative selection on mass but not on wing chord. However, the correlation between selection and size trends was more robust for wing chord than for mass, indicating the adaptive nature of these trends has yet to be confirmed.

**Table 2:** Summary of 19 studies on birds and mammals showing temporal trends in size (details in Table S1)

Order	Species	Temperature range	Genetic	Plastic	Adaptation	Causal	Time	Reference
Studies involving a single species								
Passeriformes	Great tit *(Parus major)*	1.74°C	*N* (1, 3)	*Y* (1)	*N* (2)	TP	FD	Husby et al. (2011)
Passeriformes	Dipper *(Cinclus cinclus)*	1.47°C	.	.	.	.	FD	Moreno-Rueda and Rivas (2007)
Artiodactyla	Soay sheep *(Ovis aries)*	0.35°C	*N* (3)	*Y* (5)	*N* (2)	TP	FD	Ozgul et al. (2009)
Rodentia	Yellow-bellied marmot *(Marmota flaviventris)*	0.61°C	.	*Y* (5)	*Y* (2)	TP, SM	FD	Ozgul et al. (2010)
Charadriiformes	Red-billed gull *(Chroicocephalus scopulinus)*	0.49°C	*N* (1, 3)	*Y* (1)	*N* (2)	.	FD	Teplitsky et al. (2008)
Soricomorpha	Masked shrew *(Sorex cinereus)*	2°C	.	.	.	TP (1)	MS	Yom-Tov and Yom-Tov (2005)
Carnivora	Otter *(Lutra lutra)*	0.53°C	.	.	.	FR (1)	MS	Yom-Tov et al. (2006a)
Carnivora	American marten *(Martes Americana)*	2°C	.	.	.	TP (1)	MS	Yom-Tov et al. (2008)
Carnivora	Stone marten *(Martes foina)*	0.55°C	.	.	.	TP (1)	MS	Yom-Tov et al. (2010a)
Carnivora	Otter *(Lutra lutra)*	1.8°C	.	.	.	TP (1)	MS	Yom-Tov et al. (2010b)
Studies involving more than one species								
Rodentia	3 different sp.	2.05°C	.	.	.	TP, SM (1)	MS	Eastman et al. (2012)
Passeriformes	8 different sp.	0.7°C	.	.	.	TP (1)	MS	Gardner et al. (2009)
	70 different sp.	< 0.9°C	.	.	.	TP, PR (1)	FD	Goodman et al. (2012)
Passeriformes	6 different sp.	0.94°C	.	.	.	.	MS	McCoy (2012)
Carnivora	22 different sp.	< 1°C	.	.	.	.	MS	Meiri et al. (2009)
	102 different sp.	0.7 – 1.3°C	.	.	*Y*	TP (1)	FD	Van Buskirk et al. (2010)
Passeriformes	5 different sp.	1.27°C	.	.	.	.	MS	Yom-Tov (2001)
Carnivora	2 different sp.	1°C	.	.	.	FR (1)	MS	Yom-Tov et al. (2003)
Passeriformes	13 different sp.	0.9 to 1°C	.	.	.	TP, PR (1)	FD	Yom-Tov et al. (2006b)

Primary driver (causal driver of change): NS, not specific; TP, temperature; PR, precipitation; SM, snow melt; FR, food resource; Time (time component included in data collection): MS, museum specimen; FD, field observations through time.

A ‘*Y*’ indicates that evidence was found for genetic or plastic responses in traits or that adaptability or causality was investigated; ‘*N*’ indicates evidence was not found; ‘**–**’ indicates that it was not investigated. Numbers next to a ‘*Y*’ or ‘*N*’ denote the method of investigation invoked, in cases with no numbers, a method was invoked that does not fit into one of the categories used for this review.

Genetic categories: 1 = Animal models, 2 = Common garden studies, 3 = Comparison to model predictions, 4 = Experimental evolution, 5 = Space for time substitution, 6 = Molecular genetic approaches; Plastic categories: 1 = Animal models, 2 = Common garden studies, 3 = Experimental studies, 4 = Fine-grained population responses, 5 = Individual plasticity in nature; Adaptation categories: 1 = Reciprocal transplants, 2 = Phenotypic selection estimates, 3 = Genotypic selection estimates, 4 = *Q*_st_−*F*_st_; Causal categories: 1 = Common sense, 2 = Phenotype by environment interactions, 3 = Experimental selection/evolution; For full descriptions of all categories see Merilä and Hendry (this volume).

In summary, studies reporting evidence of selection for smaller body size are still very sparse, and no overall conclusion can be drawn on the adaptive nature of temporal size trends in the context of climate warming. More importantly, more data are needed to better understand how climatic factors shape selection pressures.

### Patterns of selection on size

There is evidence that selection for body size is more often positive, but there are also many nonsignificant and negative estimates of selection on body size (Kingsolver et al. 2012). However, we know very little about temporal patterns of variation in selection (Siepielski et al. 2009; Morrissey and Hadfield 2012). Furthermore, there is very little information about selection patterns in relation to environmental factors, as it is very difficult to gain enough power to estimate selection accurately across years (Kingsolver et al. 2012). However, a couple of studies suggest that climate can be a strong driver of selection.

In their emblematic studies, Grant and Grant (2002, 2003) showed that severe drought affected availability of food type for Darwin Finches (*Geospiza fortis*) on Daphne Major in the Galápagos Islands. This shift in food type from small to hard large seeds imposed strong selection on beak shape, leading to rapid and strong evolutionary responses. Similarly, a model developed for desert birds predicted that a heat wave may select for larger body size as smaller birds will be more at risk from dehydration (McKechnie and Wolf 2010). Both Darwin's finches and desert birds represent populations evolving in extreme environments. In more temperate environments, Jiguet et al. (2006) investigated the factors underlying bird population resilience to the heat wave of 2003 in France. They found that population resilience was explained by the thermal range of the species and not by body mass. Body mass was not correlated either with the species thermal range. Although data are still scarce, it does not appear that extreme climatic events select for smaller individuals.

While models of climate change predict an increase in the frequency of extreme climatic event, the impact of less extreme but sustained long-term changes (e.g. changes in mean temperature or average rain fall), can also be important in shaping selection patterns. For example, milder winters reduced selection against small individuals in Soay sheep, which partially explained the decrease in average size (Ozgul et al. 2009). A recent study on cliff swallows *(Petrochelidon pyrrhonota)* found that normal climate variations could drive variation in selection patterns (Brown et al. 2013). In this system, stabilizing selection on tarsus length was stronger during cold, wet years, but there was no obvious increase in selection for smaller size during warm years.

### Evidence for plasticity or evolution

The nature – evolutionary or plastic – of morphological changes is important for the long-term persistence of a population (Chevin et al. 2013; Kovach-Orr and Fussmann 2013). Adaptive plasticity can be a rapid short-term response but can be limited, for example, if the environmental change exceeds the normal range of fluctuations (de Jong 2005; Ghalambor et al. 2007). Evolutionary changes imply a genetic change in the population, and such changes are now acknowledged to happen fast, on ecological time scales (Thompson 1998; Hairston et al. 2005; Carroll et al. 2007). Recently, there is increasing use of molecular methods to identify candidate genes under selection and subject to selective sweeps (Hoffmann and Willi 2008; Hansen et al. 2012). Such studies are currently scarce, but may become increasingly numerous as the cost of analyses continues to decrease for nonmodel species.

In this review, of the 19 studies showing a size trend, only two formally investigated the plastic or evolutionary basis of size change in response to climate change (Table 2).

### Is it evolution?

#### Methods

To demonstrate an evolutionary response to climate change, genetic changes in response to selection pressures triggered by climate change need to be detected. In wild populations, the animal model is a commonly used tool to assess whether phenotypic shifts have a genetic or plastic basis (Kruuk 2004). The animal model is a linear mixed model where one of the random effects is the identity of the individual, linked to its pedigree. The pedigree contains information about relatedness among individuals in the population. This approach allows disentangling the portion of phenotypic variation due to additive genetic variance from other sources of variance. The animal model also allows for the estimation of individual genetic value known as breeding values (Kruuk 2004; Wilson et al. 2010). A change in these values through time can be interpreted as a change in the genetic composition of the population. If immigration and drift can be excluded, a change in breeding values consistent with the expected change based on selection patterns can be interpreted as microevolution. Several caveats surround the use of the animal model (Postma 2006; Postma and Charmantier 2007; Hadfield et al. 2010), notably when accounting for errors in the estimation of breeding values (Hadfield et al. 2010; Wilson et al. 2010). However provided these are properly accounted for (e.g. using MCMCglmm, Hadfield 2010), the animal model remains a useful tool. Detecting temporal trends in breeding values is essential to assess whether there is an evolutionary change. Even if phenotypic selection is detected on a heritable trait, microevolutionary responses may not occur, due to genetic correlations among traits that can slow down or prevent a response to selection (Price and Langen 1992; Walsh and Blows 2009), or because selection is acting on the environmental and not on the genetic component of the trait (Price et al. 1988; Merilä et al. 2001b; Morrissey et al. 2010).

Comparison to predictions based on the direction of selection (Method 3 in Merilä and Hendry 2014) allows us, to some extent, to infer genetic changes. While a trend in the direction predicted by selection does not allow us to tease apart evolution from plasticity, the plastic nature of a change is apparent if the change occurs in the opposite direction of what is predicted by the direction of selection. In the latter case, however, a genetic change could occur but be masked by an environmental trend (Merilä et al. 2001a,b).

#### Evidence from wild populations

In great tits (Husby et al. 2011) and red billed gulls (Teplitsky et al. 2008), where a decline in body size was observed, there was no temporal change in breeding values. This led to the conclusion that phenotypic changes were due to plasticity. In both populations, plasticity seemed to be triggered by environmental degradation and was most likely nonadaptive, as there was no evidence of selection favouring smaller individuals. The absence of genetic changes in red billed gulls was expected, as no selection on size was detected. In the great tits populations, if anything, positive trends in breeding values could have been expected, as there was some evidence for positive selection on size.

In the Soay sheep (Ozgul et al. 2009), the decrease in size was not an evolutionary response, as selection on size was positive. In the case of the size increase in the yellow-bellied marmot, growth rates explained 52% of size changes, but selection explained only 3% of the variation, providing little support for an evolutionary response (Ozgul et al. 2010), although it cannot be excluded.

Under directional selection, size is likely to evolve rapidly given that body size is a complex trait with large amounts of genetic variability (e.g. Marroig and Cheverud 2010) although genetic correlations among traits may affect the pace of this response (e.g. Arnold et al. 2001; Kopp and Matuszewski 2014). Strong directional pressures on size due to selective harvesting have been shown to directly drive an evolutionary decrease in body size in bighorn sheep (*Ovis canadensis*). The selection imposed by trophy hunting targeting males with larger horns led to a decrease in horn length and body size both at the phenotypic and genetic levels, because of a strong genetic correlation between both traits (Coltman et al. 2003). Further studies on the effect of climate change on selection patterns are needed before we can speculate more on their evolutionary consequences.

### Is it plasticity?

#### Methods

An adaptive plastic response to climate change is a developmental change triggered by a change in temperature or a related climatic factor, that leads to increased fitness (see section above ‘are these trends adaptive?’). Several methods can be used to investigate phenotypic plasticity. One approach is to use of common garden experiments (also recommended by Urban and Phillips 2014), where individuals from the same population can be submitted to different environmental conditions, or the opposite (several populations, one experimental environmental conditions). There are very few examples in which the effect of temperature on body mass was directly tested in an experimental setting for mammals (see also Barnett and Dickson 1984). Riek and Geiser (2012) induced a larger size (both head length and body length) in fat-tailed dunnarts (*Sminthopsis crassicaudata*) reared in a cold environment (16°C) compared to individuals reared in a warm environment (22°C). No other experimental evidence for developmental phenotypic plasticity induced by exposure to cold has been done for birds or mammals but generally, this experiment suggests that developmental plasticity in response to temperature is possible.

When experiments are not practical, other methods are available for analysing long-term population trends in the wild. First, the animal model allows us to assess plasticity: a phenotypic trend in the absence of a genetic changes implies plasticity. A second approach is to detect fine grain responses, given that plasticity allows a rapid response that closely tracks environmental variations. One possibility is to assess if temperature explains better variation in size than time alone, as done by Charmantier et al. (2008) for laying date in great tits. In their study of the stone marten (*Martes foina*) in Denmark, Yom-Tov et al. (2010a) found that there was no overall significant size decrease over the last 150 years. A closer look at the data revealed that skull size decreased during two periods of temperature increase that were separated by a short cooling period. Despite the correlation, these size decreases were not significantly related with mean annual temperature. A third, more direct, approach is the analysis of individual plasticity by (i) using a derivation of the Price equation to directly estimate the contribution of plasticity to a trait change in a variable environment (Coulson and Tuljapurkar 2008) or (ii) using within-subject centring, a statistical procedure that can discriminate within- from between-subject effects, and can thus be used to distinguish population from individual plasticity (Van de Pol and Wright 2009). Using a similar approach, Phillimore et al. (2010) compared intra- and interpopulations responses to temperature to assess whether local adaptation or plasticity were explaining differences in spawning date among *Rana temporaria* in Britain. This approach, however, is not commonly used, because most field studies focus on within-population variability and not among-population variability.

#### Insights from wild populations

In our data set, we retrieved four studies that assessed the nature of temporal size trends (Table 2), and all concluded to an environmentally induced response. Size decreases in red billed gulls (Teplitsky et al. 2008) and great tits (Husby et al. 2011) were not due to microevolutionary change but to nonadaptive plasticity. The decline in size of Soay sheep (Ozgul et al. 2009) and increase in size of the yellow-bellied marmots (Ozgul et al. 2010) were both due to plastic responses to changing environmental conditions, mostly affecting growth rates. The decrease in size in Soay sheep was density-dependent, with increased survival in slow growing individuals and increasing population density which negatively influenced lamb growth rates (Ozgul et al. 2009). The increase in body mass in the yellow-bellied marmot resulted from a longer growing season due to shifts in the phenology of individuals (e.g. an earlier weaning of offspring and earlier emergence from hibernation, Ozgul et al. 2010).

In wild populations, it is essential to identify the factors that trigger the plastic response to understand the effects of phenotypic changes on population persistence. Many factors other than temperature can influence body size, and a phenotypic temporal trend in a locality where temperature is increasing does not provide sufficient evidence of a response to climate change. Assessing whether size trends are a response to climate change is challenging for at least two reasons.

First, climate change can affect body size through both direct (e.g. a decrease in heat loss during milder winters which allows for an increased energetic allocation towards growth) and indirect effects (e.g. shorter winter and increased length of the growing season providing a longer access to food resources). A correlation between body size and temperature may not be apparent if the temporal size trend is linked to indirect effects of climate change, but this would not rule out a response to climate change. For example, in the Dutch great tit population, the body size decline observed in the past 29 years was not correlated with temperature at the breeding grounds, but was at least partially explained by the mismatch between hatching date and peak of food abundance (caterpillars, Husby et al. 2011). This temporal mismatch increased during the study period and was a consequence of climate change. Another example of the indirect effect of climate change is the decrease in body size in polar bears (*Ursus maritimus*) between 1982 and 2006 in the Beaufort Sea, Alaska, due to the lower availability of sea ice, their optimal habitat (Rode et al. 2010). Disentangling direct and indirect effects of climate change is not straightforward and requires extensive knowledge of the study system.

Second, a correlation between size and temperature may arise because both are changing through time but without a causal link between them. In red billed gulls, the body size decrease was correlated with a temperature anomaly in New Zealand (Teplitsky et al. 2008). Survival was also declining in this population (Teplitsky et al. 2008), but the decreased survival rate did not correlate with temperature (A. Robert & J. A. Mills, unpubl. res.), suggesting that an environmental stressor other than temperature, is acting on this population. In the context of rapid global change, several possibly interacting factors (e.g. food abundance, hunting pressure) may be varying at the same time and influencing body size. Teasing apart the relative influences of different factors on body size change in natural populations can be challenging. For example, the same phenotypic response within the same species could be due to different factors among populations. In the European otter (*Lutra lutra*), both Swedish and Norwegian populations showed an increase in size through time. In the Swedish populations, this was attributed to decreased ice cover and longer fishing periods (Yom-Tov et al. 2010b), while this increase in their Norwegian conspecifics would be related with increased food availability due to fish farming (Yom-Tov et al. 2006a).

## Conclusions

To date, there is no direct evidence that body size decreases in birds and mammals are an adaptive response to climate change. Of the three studies investigating size declines using long-term individual-based data (Teplitsky et al. 2008; Ozgul et al. 2009; Husby et al. 2011), none found evidence of selection for smaller size, and instead hinted this was due to a change in environment quality. Consequently, in these cases, evolutionary change could not be expected to be the basis of phenotypic change in size. However, these three studies represent a very small amount of data relative to the number of studies reporting recent changes in size, and further investigation of the origin (plastic and/or genetic) and nature (adaptive or not) of size declines is needed. These conclusions are in line with findings for changes in phenology in birds and mammals, for which evidence for microevolution is also very scarce, although the adaptive nature of the change in phenology is more firmly established in birds (Boutin and Lane 2014; Charmantier and Gienapp 2014).

Bergmann's rule may not provide the most pertinent framework for the study of size changes in the context of climate change. In the absence of well-identified underlying mechanisms for it, Bergmann's rule is not too helpful in interpreting recent temporal size trends. Instead of focusing on whether body size changes are in agreement with Bergmann's rule (Bergmann's rule as a pattern), we need to focus on Bergmann's rule as a mechanism and identify the factors that are at the basis of size changes.

**Figure 1:**
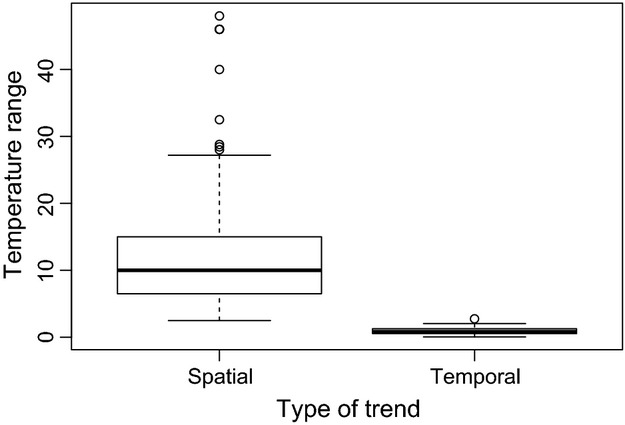
The temperature gradients (spatial versus temporal) over which size trends were quantified; Data for geographical gradients are from the reviews of Ashton et al. (2000) for mammals, and Ashton (2002) for birds. If the temperature gradient was not directly reported in the study, it was estimated from the latitudinal gradient. Data for temporal gradients are from this review. We used the global change in temperature anomaly to estimate the change in temperature when it was not readily available from the publication. On average, geographical trends were studied over a temperature gradient of 12.29**°**C, a gradient significantly larger than the recent climate warming (average temperature increase of 0.93**°**C in this review, *t*-test *P <* 0.0001).

**Figure 2:**
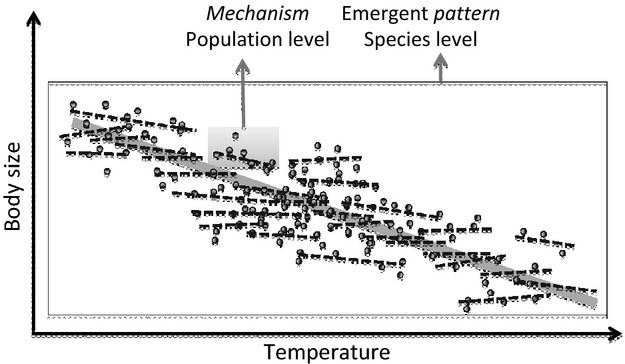
A theoretical framework for the effect of study scales on Bergmann's rule in space and time; *X*-axis: temperature observed across either a geographical gradient or a temporal gradient; *Y*-axis: body size. Each dot represents an individual. The dotted lines represent reaction norms at the population level. At the population level, there is no relation between body size and temperature, as the gradient in temperature within a population may be too low to detect such a pattern. However, individuals are still selected for an optimal temperature. Bergmann's rule, the decrease in body size with temperature is a pattern emerging at larger geographical or temporal scales.

Finally, Bergmann's rule may not hold at all geographical and temporal scales. This rule received the most support from large-scale studies. The pattern of larger body size at higher latitudes is supported by a majority of mammal and bird species, when studied across a large regional or continental scale (Ashton et al. 2000; e.g. Ashton 2002; Meiri and Dayan 2003; Millien et al. 2006). Blackburn et al. (1999), in their re-definition of Bergmann's rule, concluded that it is a pattern emerging across species, ‘in a monophyletic higher taxon’. The relation between temperature and body size change through time has also received much support at large temporal scales. There is some evidence that many mammal species decreased in body size since the last glacial maximum (e.g. Smith et al. 1995). The trend is also apparent at larger temporal scales in the fossil record (reviewed in Millien et al. 2006). Yet, as reported in this review, support for a decrease in body size over the last few decades in response to climate change is not as strong, especially in mammals (Table 1). Overall, we found that 54% of the species reviewed here decreased in size over the last few decades (60% of birds and 8% of mammals). These numbers are below what is reported for geographical trends over large spatial scales (Ashton et al. 2000; Ashton 2002; Meiri and Dayan 2003; Millien et al. 2006). Moreover, the range of temperature varies across the type of studies: Bergmann's rule in space was studied over significantly larger temperature gradients than the recent change in temperature accompanying climate warming (Fig. 1). This suggests that the *pattern* of Bergmann's rule is emerging across a minimum temperature gradient, whether this gradient operates in space or time (Fig. 2). The *mechanisms* driving Bergmann's rule, however, are necessarily operating at the individual level, assuming that Bergmann's rule through time is adaptive and that individuals following the rule are selected for. Though an intuitive assumption, we have shown here that demonstrating the adaptive or evolutionary nature of Bergmann's rule is not straightforward, partly because of the complexity of the mechanisms and interacting factors acting upon body size.

It has recently been suggested that body size decrease is a universal response to climate change (e.g. Gardner et al. 2011; e.g. Sheridan and Bickford 2011). However, the evidence we have collated here indicates that recent size declines in the context of climate warming are highly system- and context dependent. No general pattern or rule is to be expected, because organisms respond differently in terms of the direction, amount and rate of size change. Thus, the response of species interactions within communities may even be more complex. Hence, our challenge is to better understand the adaptive and evolutionary basis of these size changes before we can evaluate the resilience of ecological interactions that are affected by these size changes.

## Supporting Information

**Table S1.** Studies investigating time trends in relation to climate change and Bergmann's rule for birds and mammals.
