# Supplementary material for: Climate warming and Bergmann's rule through time: is there any evidence?
Source: Evol Appl. 2013 Nov 25;7(1):156–68. doi: 10.1111/eva.12129 (PMC3894904; doi:10.1111/eva.12129)
Supplement: Supplementary file 1 [file eva0007-0156-SD1.doc]

**Table S1**. Studies investigating time trends in relation to climate change and Bergmann’s rule for birds and mammals.

**Population:**  Both location and time range are given.

**Temperature range:**  if not provided in the text, we used the difference in the global annual mean temperature anomaly

**Fits Bergmann**: “Yes” if there is a size decline either temporal or in correlation with increased temperature, “No” otherwise. If multiple species within a study were investigated and per species details were not readily available, data were summarized by “x out of N” species showing a size decline. If more than half responded in the direction predicted by Bergmann’s rule then ‘Yes (x/N)’ if less then half showed a decline in size ‘No (x/N)’

**Genetic change:** “Yes” if a genetic change in the population was formally tested, “No” if the trend was tested but did not exist, and “.” if genetic trend was not assessed.

**Plastic change:** “Yes” if a plastic change in the population was formally tested, “No” if the trend was tested but did not exist, and “.” if plastic response was not tested.

**Adaptive:** “Yes” if selection for smaller size was found, “No” if there is evidence for no selection on size or selection for larger size and “.” if selection was not tested.

**Year effect**: slope and significance of the size against year regression model if available, otherwise conclusion about the direction and significance of year effect. If multiple species within a study were investigated and details were not readily available, data were summarized by “x out of N” species.

**Temperature** effect: slope and significance of the size against temperature regression model if available, otherwise conclusion about the direction and significance of temperature effect. If multiple species within a study were investigated and details were not readily available, data were summarized by “x out of N” species.

**Likely factor**: Potential factors other than temperature that could drive the size trend and cited in the reference. They are reported with (H): simple hypothesis, (C): correlation tested.

| Species | Population | Temperature range | Trait | Fits Bergmann | Genetic change | Plastic change | Adaptive | Year effect | Temp. effect | Likely Factor | Reference |
| --- | --- | --- | --- | --- | --- | --- | --- | --- | --- | --- | --- |
| Belding’s ground squirrel,  *Urocitellus beldingi* | Sierra Nevada, USA | (1902-1950) vs. (2000-2008) | 2.05°C | Skull length | No | . | . | . | Increase  (P<0.001) | . | Length feeding season (H) | (Eastman et al. 2012) |
|  |  |  | Maxillary toothraw length | No trend | . | . | . | none  (P=0.91) | . | . |  |
| Golden manteled ground squirrel, *Callospermophilus lateralis* | Sierra Nevada, USA |  | Skull length | No | . | . | . | Increase  (P=0.018) | . | Length feeding season (H) |  |
|  |  |  | Maxilary toothraw length | No trend | . | . | . | none  (P=0.44) | . | . |  |
| California ground squirrel, *Otospermophilus beecheyi* | Sierra Nevada, USA |  | Skull length | No trend | . | . | . | none  (P=0.22) | . | . |  |
|  |  |  | Maxilary toothraw length | No trend | . | . | . | none  (P=0.51) | . | . |  |
| Grey crowned babbler, *Pomatostomus temporalis* | South eastern Australia | 1860 to 2001, pre-1950 vs. post-1950 | 0.7°C | Wing length | Yes | . | . | . | -3.45 ± 0.88  (P<0.001) | . | Temperature (H) | (Gardner et al. 2009) |
| Jacky winter, *Microeca fascinans* |  |  |  | Yes | . | . | . | -1.53 ± 0.66  (P=0.006) | . | Temperature (H) |  |
| Hooded robin, *Melanodryas cucullata* |  |  |  | Yes | . | . | . | -3.59 ± 0.92  (P=0.002) | . | Temperature (H) |  |
| Brown treecreeper, *Climacteris picumnus* |  |  |  | Yes | . | . | . | -1.03 ± 0.63  (P=0.08) | . | Temperature (H) |  |
| White-brown scrubwren, *Sericornis frontalis* |  |  |  | No trend | . | . | . | none  (P=0.54) | . | Temperature (H) |  |
| Variegated fairy-wren, *Malurus lamberti* |  |  |  | Yes | . | . | . | -1.08 ± 0.70  (P=0.056) | . | Temperature (H) |  |
| Yellow-rumped thornbill, *Acanthiza chrysorrhoa* |  |  |  | Yes | . | . | . | -2.08 ± 0.654  (P=0.003) | . | Temperature (H) |  |
| Speckled warbler, *Pyrrholaemus sagittatus* |  |  |  | No trend | . | . | . | none  (P=0.71) | . | Temperature (H) |  |
| 41 bird species (Summer) | Palomarin, California, USA | 1971 - 2010 | 0.54 | Body Mass | No | . | . | . | 0.0004 ± 0.00026 g/year (P <0.0027) | . | Climate variability / increased productivity (H) | (Goodman et al. 2012) |
|  |  |  | Wing length | No | . | . | . | 0.00051 ± 0.0001 mm/year(P <0.001) | . |  |  |
| 26 bird species (Winter) |  |  | Body Mass | No trend | . | . | . | none (P=0.11) | . |  |  |
|  |  |  | Wing length | No | . | . | . | 0.00039 ± 0.00025 mm/year (P <0.0021) | . |  |  |
| 45 bird species (Spring) |  |  | Wing length | No | . | . | . | 0.00025 ± 0.00018 mm/year (P<0.007) | . |  |  |
| 35 bird species (Fall) |  |  | Body Mass | No trend | . | . | . | none (P=0.23) | . |  |  |
| 23 bird species (Summer) | Coyote Creek, California, USA | 1983 - 2009 | 0.80 | Body mass | No | . | . | . | 0.00112 ± 0.00109 g/year (P<0.022) | . |  |  |
|  |  |  | Wing length | No | . | . | . | 0.00057 ± 0.00034 mm/year (P<0.056) | . |  |  |
| 20 bird species (Winter) |  |  | Body mass | No trend | . | . | . | none (P=0.47) | . |  |  |
|  |  |  | Wing length | No | . | . | . | 0.00056 ± 0.00025 mm/year (P<0.058) | . |  |  |
| 38 bird species (Spring) |  |  | Body mass | No | . | . | . | 0.00096 ± 0.00059 g/year (P<0.0015) | . |  |  |
|  |  |  | Wing length | No | . | . | . | 0.00058 ± 0.00021 mm/year (P<0.0001) | . |  |  |
| 26 bird species (Fall) |  |  | Wing length | No | . | . | . | 0.00084 ± 0.00029 mm/year (P<0.0001) | . |  |  |
| Great tits, *Parus major* | Hoge Veluwe, Netherlands | 1979-2008 | 1.74°C | Body mass | Yes | No | Yes | No | -0.013 ± 0.004 g/year  (P<0.01) | March-February (P=0.35)  Yearly mean (P=0.06) | Synchrony with food peak (C)  0.59 ± 0.19  (P=0.006) | (Husby et al. 2011) |
|  |  |  | Tarsus length | No trend | No | Yes | No | none (P>0.05) | P >0.14 |  |  |
|  | Oosterhout, Netherlands \| 1979-2008 |  | Body mass | Yes | No | Yes | No | -0.012 ± 0.006 g/year  (P<0.05) | . | . |  |
|  |  |  | Tarsus length | No | No | Yes | No | -0.006 ±0.006 mm/year  (P=0.05) | . | . |  |
|  | Vlieland, Netherlands \| 1979-2008 |  | Body mass | Yes | No | Yes | No | -0.015 ± 0.005 g/year  (P<0.01) | . | . |  |
|  |  |  | Tarsus length | No | No | Yes | No | 0.0126 ± 0.004 mm/year  (P<0.01) | . | . |  |
| Merriam’s kangaroo rat, *Dipodomys merriami* | Sevilleta, USA | 1989-1996 | 2.5 to 3°C | Body mass | No trend | . | . | . | none | none | . | (Koontz et al. 2001) |
| Purple Finch, *Carpodacus purpureus* | Connecticut, USA| 1874-1952 vs. 1958-2010 | 0.94°C (SD = 0.71) | Wing length | Yes | . | . | . | -2.4% (P<0.00025) | . | . | (McCoy 2012) |
| Blue Jay, *Cyanocitta cristata* |  |  |  | Yes | . | . | . | -3.2% (P<0.025) | . | . |  |
| House Sparrow, *Passer domesticus* |  |  |  | No trend | . | . | . | none (P=0.4) | . | . |  |
| Black-capped Chickadee, *Poecile atricapillus* |  |  |  | No trend | . | . | . | none (P=0.1) | . | . |  |
| Common Grackle, *Quiscalus quiscula* |  |  |  | Yes | . | . | . | -6.7 % (P<0.0025) | . | . |  |
| White-breasted Nuthatch, *Sitta carolinensis* |  |  |  | No trend | . | . | . | none | . | . |  |
| Arctic fox, *Alopex lagopus* | Alaska | 1897-1993 | 0.05 – 0.93°C | Condylo-basal length | No trend | . | . | . | none (P=0.435) | . | . | (Meiri et al. 2009) |
| Coyote, *Canis latrans* | Baja California \| 1884-1979 |  |  | No trend | . | . | . | none (P=0.118) | . | . |  |
| Gray wolf, *Canis lupus* | Alaska \| 1901-1994 |  |  | No trend | . | . | . | none (P=0.427) | . | . |  |
|  | British Columbia \| 1891-1982 |  |  | No trend | . | . | . | none (P=0.11) | . | . |  |
|  | Vancouver Island \| 1937-1985 |  |  | No trend | . | . | . | none (P=0.801) | . | . |  |
| Canada lynx, *Lynx canadensis* | Alaska \| 1905-1992 |  |  | No trend | . | . | . | none (P=0.258) | . | . |  |
|  | British Columbia \| 1889-1983 |  |  | No trend | . | . | . | none (P=0.361) | . | . |  |
| Wolverine, *Gulo gulo* | Alaska \| 1891-1993 |  |  | No trend | . | . | . | none (P=0.661) | . | . |  |
| American marten, *Martes americana* | Alaska \| 1860-1996 |  |  | No trend | . | . | . | none (P=0.488) | . | . |  |
|  | British Columbia \| 1889-1996 |  |  | No trend | . | . | . | none (P=0.221) | . | . |  |
|  | Vancouver Island \| 1904-1988 |  |  | No trend | . | . | . | none (P=0.672) | . | . |  |
| Beech marten, *Martes foina* | Germany \| 1876-1999 |  |  | No trend | . | . | . | none (P=0.86) | . | . |  |
| European pine marten, *Martes martes* | Benelux \| 1947-2004 |  |  | No trend | . | . | . | none (P=0.317) | . | . |  |
| Japanese marten, *Martes melampus* | Honshu \| 1944-1991 |  |  | No trend | . | . | . | none (P=0.247) | . | . |  |
| European badger, *Meles meles* | Benelux \| 1918-2000 |  |  | No trend | . | . | . | none (P=0.143) | . | . |  |
|  | Britain \| 1911-1989 |  |  | No trend | . | . | . | none (P=0.3) | . | . |  |
| Stoat, *Mustela erminea* | Alaska \| 1879-1998 |  |  | No trend | . | . | . | none (P=0.211) | . | . |  |
|  | Benelux \| 1926-1988 |  |  | Yes | . | . | . | -0.192 (P=0.015) | . | . |  |
|  | Britain \| 1890-1984 |  |  | No | . | . | . | 0.171 (P<0.0001) | . | . |  |
|  | British Columbia \| 1891-1999 |  |  | No trend | . | . | . | none (P=0.794) | . | . |  |
|  | Germany \| 1887-1994 |  |  | No trend | . | . | . | none (P=0.536) | . | . |  |
|  | Ireland \| 1895-1982 |  |  | No | . | . | . | 0.124 (P=0.028) | . | . |  |
|  | Labrador \| 1898-1990 |  |  | No trend | . | . | . | none (P=0.963) | . | . |  |
|  | Minnesota \| 1890-1980 |  |  | Yes | . | . | . | -0.239 (P<0.0001) | . | . |  |
|  | New England \| 1874-1997 |  |  | No trend | . | . | . | none (P=0.143) | . | . |  |
|  | Ontario \| 1893-1981 |  |  | No trend | . | . | . | none (P=0.258) | . | . |  |
|  | Washington \| 1894-1974 |  |  | No trend | . | . | . | none (P=0.243) | . | . |  |
| Long-tailed weasel, *Mustela frenata* | British Columbia \| 1894-1999 |  |  | No trend | . | . | . | none (P=0.921) | . | . |  |
|  | California \| 1885-1974 |  |  | No trend | . | . | . | none (P=0.445) | . | . |  |
|  | Michigan \| 1903-1971 |  |  | No trend | . | . | . | none (P=0.058) | . | . |  |
|  | New England \| 1864-1979 |  |  | Yes | . | . | . | -0.171 (P=0.003) | . | . |  |
|  | Oregon \| 1883-1990 |  |  | No trend | . | . | . | none (P=0.19) | . | . |  |
|  | Washington \| 1891-1960 |  |  | No trend | . | . | . | none (P=0.138) | . | . |  |
| Least weasel, *Mustela nivalis* | Alaska \| 1900-1987 |  |  | No trend | . | . | . | none (P=0.235) | . | . |  |
|  | Benelux \| 1928-1990 |  |  | No trend | . | . | . | none (P=0.137) | . | . |  |
|  | Britain \| 1895-1987 |  |  | No trend | . | . | . | none (P=0.895) | . | . |  |
|  | Italy \| 1883-1972 |  |  | No trend | . | . | . | none (P=0.74) | . | . |  |
| European polecat, *Mustela putorius* | Benelux \| 1912-2004 |  |  | No trend | . | . | . | none (P=0.906) | . | . |  |
| American mink, *Mustela vison* | Alaska \| 1904-1999 |  |  | No | . | . | . | 0.361 (P<0.0001) | . | . |  |
|  | Ontario \| 1912-1955 |  |  | No trend | . | . | . | none (P=0.722) | . | . |  |
|  | Vancouver Island \| 1886-1975 |  |  | No trend | . | . | . | none (P=0.224) | . | . |  |
| Raccoon dog, *Nyctereutes procyonoides* | Honshu \| 1949-1989 |  |  | No trend | . | . | . | none (P=0.421) | . | . |  |
| Raccoon, *Procyon lotor* | Florida \| 1891-1999 |  |  | No trend | . | . | . | none (P=0.375) | . | . |  |
| Western spotted skunk, *Spilogale gracilis* | California \| 1887-1954 |  |  | No trend | . | . | . | none (P=0.579) | . | . |  |
| Gray fox, *Urocyon cinereoargenteus* | California \| 1891-1987 |  |  | No trend | . | . | . | none (P=0.657) | . | . |  |
| Brown bear, *Ursus arctos* | Admiralty Island \| 1905-1979 |  |  | No trend | . | . | . | none (P=0.824) | . | . |  |
|  | Alaska \| 1894-1975 |  |  | No trend | . | . | . | none (P=0.103) | . | . |  |
| Oriental civet, *Viverra tangalunga* | Borneo \| 1887-1962 |  |  | No trend | . | . | . | none (P=0.8) | . | . |  |
| Red fox, *Vulpes vulpes* | Alaska \| 1903-1996 |  |  | No trend | . | . | . | none (P=0.263) | . | . |  |
|  | Benelux \| 1932-2001 |  |  | No trend | . | . | . | none (P=0.187) | . | . |  |
|  | France \| 1909-1992 |  |  | No trend | . | . | . | none (P=0.052) | . | . |  |
|  | Israel \| 1945-2000 |  |  | No trend | . | . | . | none (P=0.079) | . | . |  |
| Dipper,  *(Cinclus cinclus)* - male | Sierra Nevada, Spain | 1985-2005 | 1.47°C (se = 0.63) | Tarsus length | Yes | . | . | . | Decrease (P<0.01) | . | . | (Moreno-Rueda and Rivas 2007) |
|  |  |  | Wing length | No | . | . | . | Increase (P<0.01) | . | . |  |
|  |  |  | Tail length | No | . | . | . | Increase (P<0.01) | . | . |  |
|  |  |  | Body mass | No trend | . | . | . | none (P>0.05) | . | . |  |
|  |  |  | Beak | No trend | . | . | . | none (P>0.05) | . | . |  |
| Dipper,  *(Cinclus cinclus)* - female |  |  | Tarsus length | No trend | . | . | . | none (P>0.05) | . | . |  |
|  |  |  | Wing length | No trend | . | . | . | none (P>0.05) | . | . |  |
|  |  |  | Tail length | No trend | . | . | . | none (P>0.05) | . | . |  |
|  |  |  | Body mass | No trend | . | . | . | none (P>0.05) | . | . |  |
|  |  |  | Beak | No trend | . | . | . | none (P>0.05) | . | . |  |
| Soay sheep, *Ovis aries* | St Kilda, Scotland | 1986-2006 | 0.35 °C | Body mass | Yes | . | Yes | No | -81g/year | . | Shorter and milder winters (C) | (Ozgul et al. 2009) |
| Yellow bellied marmot, *Marmota flaviventris* | Colorado, USA | 1976-2008 | 0.61 °C | Body mass | No | . | Yes | No | Increase | . | Earlier phenology (C) | (Ozgul et al. 2010) |
| Red-billed gull, *Chroicocephalus scopulinus* | Kaikoura, New Zealand | 1958-2004 | 0.49 °C | Body mass | Yes | No | Yes | No | -0.28 ± 0.05 (P<0.001) | -3.57 ± 1.98 (P=0.032) | Stress (H) | (Teplitsky et al. 2008) |
| 61 breeding bird species | Pennsylvania, USA | 1961-2006 | 0.7515°C | Fat free mass | Yes (51/61) | . | . | . | Decrease |  |  | (Van Buskirk et al. 2010) |
|  |  |  | Wing chord | No trend | . | . | . | None |  |  |  |
| 26 wintering bird species |  |  | Fat free mass | Yes (20/26) | . | . | . | Decrease |  |  |  |
|  |  |  | Wing chord | No (0/26) | . | . | . | Increase |  |  |  |
| 83 spring migrant bird species |  |  | Fat free mass | Yes (60/83) | . | . | Likely | Decrease |  |  |  |
|  |  |  | Wing chord | Yes (60/83) | . | . | Likely | Decrease |  |  |  |
| 75 autumn migrants bird species |  |  | Fat free mass | Yes (66/75) | . | . | . | Decrease |  |  |  |
|  |  |  | Wing chord | Yes (52/75) | . | . | . | Decrease |  |  |  |
| Crested lark, *Galerida cristata* | Israel | 1950-1999 | 1.27°C | Body mass | No |  |  |  | none (P=0.29) |  |  | (Yom-Tov 2001) |
|  |  |  | Tarsus length | No trend |  |  |  | none (P=0.46) |  |  |  |
| House sparrow, *Passer domesticus* |  |  | Body mass | Yes |  |  |  | -13.4% (P=0.032) |  | Temperature (H) |  |
|  |  |  | Tarsus length | Yes |  |  |  | -8.7% (P=0.011) |  | Temperature (H) |  |
| Graceful warbler, *Prinia gracilis* |  |  | Body mass | Yes |  |  |  | -26.8% (P=0.006) |  | Temperature (H) |  |
|  |  |  | Tarsus length | Yes |  |  |  | -15.6% (P=0.001) |  | Temperature (H) |  |
| Yellow vented bulbul, *Pycnonotus xantophygos* |  |  | Body mass | Yes |  |  |  | -14% (P=0.007) |  | Temperature (H) |  |
|  |  |  | Tarsus length | No trend |  |  |  | none (P=0.37) |  |  |  |
| Sardinian warbler, *Sylvia melanocephala* |  |  | Body mass | Yes |  |  |  | -27.2% (P=0.003) |  | Temperature (H) |  |
|  |  |  | Tarsus length | No trend |  |  |  | none (P=0.081) |  |  |  |
| Chukar partridges, *Alectoris chukar* | Israel | Second half of the 20^th^ century | 1.3°C | Body mass | Yes |  |  |  | -0.41 ± 0.17 (P=0.015) | Jan Temp 5.9±1.6 (P<0.001)  Aug Temp -10.4±1.8 (P<0.001) | Multiple factors correlated with latitude (H) | (Yom-Tov et al. 2002) |
|  |  |  | Tarsus length | No trend |  |  |  | none (P=0.12) | Jan Temp 0.8±0.15 (P<0.001)  Aug Temp -0.6±0.17 (P<0.001) |  |  |
| Red fox, *Vulpes vulpes* | Denmark, Zealand and Jutland rural areas | 1900-2000 | 1°C | Skull length | No trend | . | . | . | none | none (P=0.68) | Diet (H) | (Yom-Tov et al. 2003) |
|  |  |  | Zygomatic breadth | No | . | . | . | 0.0385 (P<0.001) | None (P=0.41) |  |  |
|  |  |  | P4 length | No trend | . | . | . | none | Increase (P<0.0034) |  |  |
|  |  |  | Canine diameter | No | . | . | . | 0.0046 (P<0.001) | none (P=0.05) |  |  |
| Eurasian badger, *Meles meles* |  |  | Skull size | No trend | . | . | . | none | none (P=0.96) |  |  |
|  |  |  | Zygomatic breadth | No trend | . | . | . | none | none (P=0.66) |  |  |
|  |  |  | P4 length | No trend | . | . | . | none | none (P=0.19) |  |  |
|  |  |  | Canine diameter | No trend | . | . | . | none | none (P=0.11) |  |  |
| Masked shrew, *Sorex cinereus* | Alaska, USA | 1950-2003 | 2°C | Body mass | No trend | . | . | . | none (P=0.62) | 0.045 (P=0.002) | Diet (H) | (Yom-Tov and Yom-Tov 2005) |
|  |  |  | Body length | No | . | . | . | 0.308 (P<0.001) | 0.588 (P<0.001) |  |  |
|  |  |  | Tail length | No | . | . | . | 0.145 (P=0.002) | 0.345 (P<0.001) |  |  |
|  |  |  | Hind foot length | No trend | . | . | . | none (P=0.83) | 0.052 (P<0.001) |  |  |
| Otter, *Lutra lutra* | Norway | 1975-2004 | 0.53°C | Skull size (Zygomatic breadth) | No | . | . | . | 0.046 (P<0.001) | . | Fish production (C) 0.466 (P=0.001) | (Yom-Tov et al. 2006a) |
| Bullfinch, *Pyrrhula pyrrhula* | Wicken Fen, Cambridgeshire, UK | 1968-2003 | 0.86°C | Body mass | Yes | . | . | . | -0.014 (P=0.005) | . | Temperature (H) | (Yom-Tov et al. 2006b) |
| Reed warblers, *Acrocephalus scirpaceus* |  |  |  | Yes | . | . | . | 0.007year -0.001 year² (P=0.012) |  | Temperature (H) and “additional selection pressure” (not identified) |  |
| Blackcaps, *Sylvia atricapilla* |  |  |  | Yes | . | . | . | 0.014year-0.001year² (P=0.012) |  |  |  |
| Blackbirds, *Turdus merula* |  |  |  | No | . | . | . | 0.013 (P=0.024) |  | Rainfall and Diet (H) |  |
| Bullfinch, *Pyrrhula pyrrhula* |  |  | Wing length | No | . | . | . | 0.033 (P<0.001) |  | Allen’s rule: increase in the size of appendages with temperature (H) |  |
| Reed buntings, *Emberiza schoeniclus* |  |  |  | No | . | . | . | 0.076 (P<0.001) |  |  |  |
| Reed warblers, *Acrocephalus scirpaceus* |  |  |  | No | . | . | . | 0.059 (P<0.001) |  |  |  |
| Blackcaps, *Sylvia atricapilla* |  |  |  | No | . | . | . | 0.049 (P<0.001) |  |  |  |
| Blackbirds, *Turdus merula* |  |  |  | No | . | . | . | 0.064 (P<0.001) |  |  |  |
| Robins, *Erithacus rubecula* |  |  |  | No | . | . | . | 0.057 (P<0.001) |  |  |  |
| Dunnocks, *Prunella modularis* |  |  |  | No | . | . | . | 0.05 (P<0.001) |  |  |  |
| Willow warblers, *Phylloscopus trochilus* |  |  |  | Yes | . | . | . | -0.056 (P=0.02) |  |  |  |
| Blue tits, *Cyanistes caeruleus* | Tresswell Wood, Nottinghamshire, UK | 1973-2003 | 1.02°C | Body mass | Yes | . | . | . | -0.014 (P<0.001) |  | Temperature (H) |  |
| Great tits*, Parus major* |  |  |  | Yes | . | . | . | -0.036 (P<0.001) |  | Temperature (H) |  |
| Dunnocks, *Prunella modularis* |  |  |  | Yes | . | . | . | -0.013 (P=0.008) |  | Temperature (H) |  |
| Blackbirds, *Turdus merula* |  |  | Wing length | No | . | . | . | 0.028 year +0.003 year² (P=0.034) |  | Allen’s rule and additional factors (not identified) (H) |  |
| Dunnocks, *Prunella modularis* |  |  |  | No | . | . | . | 0.013year + 0.003 year² (P=0.001) |  |  |  |
| Wrens, ***Troglodytes troglodytes*** |  |  |  | No |  |  |  | 0.008year + 0.003 year² (P=0.001) |  |  |  |
| Blue tits, *Cyanistes caeruleus* |  |  |  | Yes | . | . | . | -0.022 (P=0.002) |  |  |  |
| Great tits, *Parus major* |  |  |  | Yes | . | . | . | -0.020 (P=0.021) |  |  |  |
| Chaffinch, *Fringilla coelebs* |  |  |  | Yes | . | . | . | -0.034 (P=0.005) |  |  |  |
| American marten, *Martes americana* | Alaska | 2°C | Skull size | No | . | . | . | Increase (P<0.001) | . | Diet (H) | (Yom-Tov et al. 2008) |
| Stone marten, *Martes foina* | Denmark | 1858-1942 | 0.7°C | PC1 (Skull length, zygomatic breadth, P4 length, canine diameter) | Yes | . | . | . | Decrease (P=0.0065) | none (P>0.21) | Temperature or diet (H) | (Yom-Tov et al. 2010a) |
|  | Denmark \| 1958-1999 | 0.55°C |  | Yes | . | . | . | Decrease (P=0.0226) | none (P>0.18) |  |  |
| Eurasian otter, *Lutra lutra* | Sweden | 1962-2008 | 1.8°C | Skull size | No | . | . | . | +3.5% (P=0.007) | Increase (P<0.001) | Effect of Ice coverage (P<0.001)  Increased food availability (H) | (Yom-Tov et al. 2010b) |
|  |  |  | Body mass | No | . | . | . | +32.2% (P<0.001) | Increase (P<0.001) |  |  |

**References in Table:**

Eastman, L. M., T. L. Morelli, K. C. Rowe, C. J. Conroy, and C. Moritz. 2012. Size increase in high elevation ground squirrels over the last century. Global Change Biology 18:1499-1508.

Gardner, J. L., R. Heinsohn, and L. Joseph. 2009. Shifting latitudinal clines in avian body size correlate with global warming in Australian passerines. Proceedings of the Royal Society B-Biological Sciences 276:3845-3852.

Goodman, R. E., G. Lebuhn, N. E. Seavy, T. Gardali, and J. D. Bluso-Demers. 2012. Avian body size changes and climate change: warming or increasing variability? Global Change Biology 18:63-73.

Husby, A., S. M. Hille, and M. E. Visser. 2011. Testing Mechanisms of Bergmann's Rule: Phenotypic Decline but No Genetic Change in Body Size in Three Passerine Bird Populations. American Naturalist 178:202-213.

Koontz, T. L., U. L. Shepherd, and D. Marshall. 2001. The effects of climate change on Merriam's kangaroo rat, Dipodomys merriami. Journal of Arid Environments 49:581-591.

McCoy, D. E. 2012. Connecticut Birds and Climate Change: Bergmann's Rule in the Fourth Dimension. Northeastern Naturalist 19:323-334.

Meiri, S., D. Guy, T. Dayan, and D. Simberloff. 2009. Global change and carnivore body size: data are stasis. Global Ecology and Biogeography 18:240-247.

Moreno-Rueda, G., and J. M. Rivas. 2007. Recent changes in allometric relationships among morphological traits in the dipper (Cinclus cinclus). Journal of Ornithology 148:489-494.

Ozgul, A., D. Z. Childs, M. K. Oli, K. B. Armitage, D. T. Blumstein, L. E. Olson, S. Tuljapurkar et al. 2010. Coupled dynamics of body mass and population growth in response to environmental change. Nature 466:482-485.

Ozgul, A., S. Tuljapurkar, T. G. Benton, J. M. Pemberton, T. H. Clutton-Brock, and T. Coulson. 2009. The dynamics of phenotypic change and the shrinking sheep of St. Kilda. Science 325.

Salewski, V., W. M. Hochachka, and W. Fiedler. 2010. Global warming and Bergmann's rule: do central European passerines adjust their body size to rising temperatures? Oecologia 162:247-260.

Teplitsky, C., J. A. Mills, J. S. Alho, J. W. Yarrall, and J. Merila. 2008. Bergmann's rule and climate change revisited: Disentangling environmental and genetic responses in a wild bird population. Proceedings of the National Academy of Sciences of the United States of America 105:13492-13496.

Van Buskirk, J., R. S. Mulvihill, and R. C. Leberman. 2010. Declining body sizes in North American birds associated with climate change. Oikos 119:1047-1055.

Yom-Tov, Y. 2001. Global warming and body mass decline in Israeli passerine birds. Proceedings of the Royal Society of London (B) 268:947-952.

Yom-Tov, Y., Y. Benjamini, and S. Kark. 2002. Global warming, Bergmann's rule and body mass - are they related? The chukar partridge (Alectoris chukar) case. Journal of Zoology 257:449-455.

Yom-Tov, Y., T. M. Heggberget, O. Wiig, and S. Yom-Tov. 2006a. Body size changes among otters, *Lutra lutra*, in Norway: the possible effects of food availability and global warming. Oecologia 150:155-160.

Yom-Tov, Y., N. Leader, S. Yom-Tov, and H. J. Baagoe. 2010a. Temperature trends and recent decline in body size of the stone marten *Martes foina* in Denmark. Mammalian Biology 75:146-150.

Yom-Tov, Y., A. Roos, P. Mortensen, O. Wiig, S. Yom-Tov, and T. M. Heggberget. 2010b. Recent changes in body size of the Eurasian otter *Lutra lutra* in Sweden. Ambio 39:496-503.

Yom-Tov, Y., and J. Yom-Tov. 2005. Global warming, Bergmann's rule and body size in the masked shrew *Sorex cinereus* Kerr in Alaska. Journal of Animal Ecology 74:803-808.

Yom-Tov, Y., S. Yom-Tov, and H. Baagoe. 2003. Increase of skull size in the red fox (Vulpes vulpes) and Eurasian badger (Meles meles) in Denmark during the twentieth century: an effect of improved diet? Evolutionary Ecology Research 5:1037-1048.

Yom-Tov, Y., S. Yom-Tov, and G. Jarrell. 2008. Recent increase in body size of the American marten Martes americana in Alaska. Biological Journal of the Linnean Society 93:701-707.

Yom-Tov, Y., S. Yom-Tov, J. Wright, C. J. R. Thorne, and R. Du Feu. 2006b. Recent changes in body weight and wing length among some British passerine birds. Oikos 112:91-101.
